# Correction: Synthesis, characterization and electrochemical analysis of cysteine modified polymers for corrosion inhibition of mild steel in aqueous 1 M HCl

**DOI:** 10.1039/c9ra90012h

**Published:** 2019-02-19

**Authors:** Mohammad A. Jafar Mazumder

**Affiliations:** Chemistry Department, King Fahd University of Petroleum and Minerals Dhahran 31261 Saudi Arabia jafar@kfupm.edu.sa +966-13-860-4277 +966-13-860-7836

## Abstract

Correction for ‘Synthesis, characterization and electrochemical analysis of cysteine modified polymers for corrosion inhibition of mild steel in aqueous 1 M HCl’ by Mohammad A. Jafar Mazumder *et al.*, *RSC Adv.*, 2019, **9**, 4277–4294.

The author regrets that the funding information was incorrectly shown in the acknowledgements section of the original manuscript. The corrected funding acknowledgement is as shown below.

The author gratefully acknowledges the research facilities provided by King Fahd University of Petroleum and Minerals (KFUPM) and the financial assistance of the Deanship of Scientific Research, KFUPM, Saudi Arabia through Internal project (# IN131047).

The Royal Society of Chemistry apologises for these errors and any consequent inconvenience to authors and readers.

## Supplementary Material

